# Preservation of Pancreatic Function Should Not Be Disregarded When Performing Pancreatectomies for Pancreatoblastoma in Children

**DOI:** 10.3390/pediatric16020033

**Published:** 2024-05-13

**Authors:** Traian Dumitrascu

**Affiliations:** Division of Surgical Oncology, Fundeni Clinical Institute, Department of General Surgery, Carol Davila University of Medicine and Pharmacy, Fundeni Street no. 258, 022328 Bucharest, Romania; traian.dumitrascu@umfcd.ro; Tel./Fax: +40-21-318-04-17

**Keywords:** pancreatoblastoma, pancreatectomy, central pancreatectomy, pancreatic exocrine insufficiency, diabetes, survival, children

## Abstract

Complete surgical resection in the context of a multimodal approach has been associated with excellent long-term survival in children diagnosed with pancreatoblastoma (PB). Traditionally, curative intent surgery for PB implies standard pancreatic resections such as pancreaticoduodenectomies and distal pancreatectomies with splenectomies, surgical procedures that may lead to significant long-term pancreatic functional deficiencies. Postoperative pancreatic functional deficiencies are particularly interesting to children because they may interfere with their development, considering their long life expectancy and the significant role of pancreatic functions in their nutritional status and growth. Thus, organ-sparing pancreatectomies, such as spleen-preserving distal pancreatectomies and central pancreatectomies, are emerging in specific tumoral pathologies in children. However, data about organ-sparing pancreatectomies’ potential role in curative-intent PB surgery in children are scarce. Based on the literature data, the current review aims to present the early and late outcomes of pancreatectomies in children (including long-term deficiencies and their potential impact on the development and quality of life), particularly for PB, and further explore the potential role of organ-sparing pancreatectomies for PB. Organ-sparing pancreatectomies are associated with better long-term pancreatic functional outcomes, particularly central pancreatectomies, and have a reduced impact on children’s development and quality of life without jeopardizing their oncological safety. The long-term preservation of pancreatic functions should not be disregarded when performing pancreatectomies for PB in children. A subset of patients with PB might benefit from organ-sparing pancreatectomies, particularly from central pancreatectomies, with the same oncological results as standard pancreatectomies but with significantly less impact on long-term functional outcomes.

## 1. Introduction

Pancreatic tumors in children are a heterogeneous but also uncommon pathology, with different histologies and prognoses compared with adults. Pancreatoblastoma (PB) appears to be the most frequent type of pancreatic malignancy in children, as a few studies based mainly on national or international registries of childhood tumors have shown [1,2,3,4,5,6,7,8,9,10,11]. However, even in the registries, the number of reported children with PB is minimal, ranging between 4 and 22 cases, covering periods from 8 to 43 years [1,3,5,6,11]. A systematic review of the literature published in 2018 identified only 81 children diagnosed with PB based on a PubMed database search [8]. A previous literature review published in 2004 identified 153 patients with PB (89% children and 11% adults) [12].

**Figure 1:**
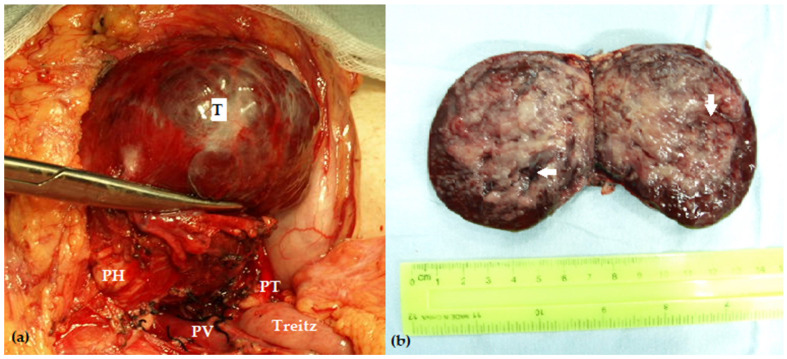
(**a**) Intraoperative aspects showing a large, relatively exophytic, encapsulated mass (T) into the pancreatic body, compressing but without invading the portal vein (PV); (**b**) the cut surface of the operative specimen (central pancreatectomy for pancreatoblastoma) showing an encapsulated heterogenous mass with cystic and hemorrhagic components (white arrows) (PH—pancreatic head; PT—pancreatic tail).

**Figure 2:**
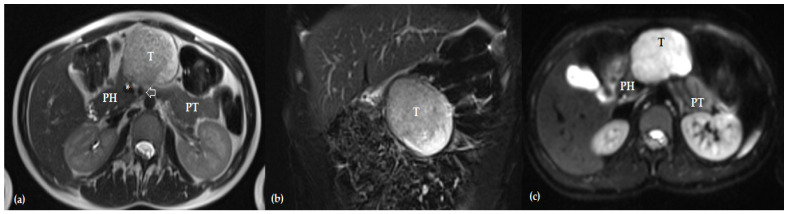
(**a**) Axial and (**b**) coronal T2-weighted magnetic resonance images showing a large, relatively exophytic, well-circumscribed mass in the pancreatic body (T) that is heterogeneously hyperintense relative to the nearby pancreatic parenchyma, compressing but without invading the portal vein (*) or superior mesenteric artery (white arrow); (**c**) axial diffusion-weighted magnetic resonance imaging showing the mass in the pancreatic body (T) with restricted diffusion compared with the nearby pancreatic parenchyma (PH—pancreatic head; PT—pancreatic tail).

PB is an embryonal tumor originating from pluripotent pancreatic stem cells during the gestational development of foregut structures; its molecular pathogenesis resembles hepatoblastoma [13]. PB is typically diagnosed in the first decade of life, while a few cases were reported in older children [8,11,13,14]. Usually, PB is a soft, large, circumscribed, encapsulated tumor (Figure 1). The diagnosis is suspected mainly at imaging examinations; PB is often heterogeneous, with solid and cystic/necrotic areas [13,15] (Figure 2). A PB is developed in the pancreatic body or tail of the pancreas in 57.9% to 64.3% of cases, at a median age of 5.3 years, with boys predominating (54.6% to 63% of cases) [8,11,16].

Considering PB’s rarity, such patients have no standardized diagnostic and therapeutic guidelines [6,13]. However, as for the most significant part of pancreatic tumors, resection (i.e., pancreatectomy) represents the single hope for long-term survival in children diagnosed with PB [8,9,10,11,13,14]. Most children resected for PB include standard pancreatic resections such as distal spleno-pancreatectomies or pancreaticoduodenectomies [8,10,13,14]. In this context, a complex surgical procedure such as pancreaticoduodenectomies is uncommonly performed in children [17].

Both distal spleno-pancreatectomies and pancreaticoduodenectomies are complex multi-visceral surgical procedures not only associated with high early morbidity rates but also with potentially significant long-term functional deficiencies. The long-term functional consequences of standard pancreatectomies are particularly interesting to children because they may interfere with their development, considering the longer life expectancy of children after pancreatectomies than adults and the significant role of pancreatic functions on their nutritional status and growth. Thus, organ-sparing pancreatectomies, such as spleen-preserving distal pancreatectomies or central pancreatectomies, are emerging in specific tumoral pathologies in children, particularly for solid pseudopapillary tumors (SPT) [8,18,19,20,21,22,23,24,25,26]. The role of organ-sparing pancreatectomies is to preserve spleen function with spleen-preserving distal pancreatectomies and spleen and pancreatic functions with central pancreatectomies. Thus, with organ-sparing pancreatectomies, there is a potential for better long-term functional outcomes and a reduced impact on children’s development and quality of life when long-term survival after pancreatectomies is expected. However, data about organ-sparing pancreatectomies’ potential role in curative-intent PB surgery in children are scarce [8,13].

Because pancreatic malignancies are rare in children, there is limited experience with this pathology, and many pediatric surgeons are unfamiliar with the biological behavior of different types of pancreatic malignancies (mainly PB), surgical approaches, and the long-term outcomes after resection. Furthermore, the main goal for a pediatric surgeon is to perform an oncologically safe pancreatectomy. In contrast, the potential detrimental effect on long-term outcomes of pancreatic function deficiencies after pancreatectomies in children is frequently disregarded. Recently, it was suggested that managing such rare pathologies in children should be performed in centers with extensive experience with pancreatectomies in adults [27]. Based on the literature data, the current narrative review aims to present the early and late outcomes of pancreatectomies in children (including long-term deficiencies and their potential impact on the development and quality of life), particularly for PB, and further explore the potential role of organ-sparing pancreatectomies for PB.

## 2. Morbidity of Standard and Organ-Sparing Pancreatectomies in Children with Pancreatic Tumors Other than PB

SPT represents the most frequent type of pancreatic tumor resected in children [4,8,9,13,22,25,27,28,29,30,31,32,33,34]. Thus, a large part of the data regarding the morbidity and long-term outcomes after pancreatectomies in children comes from a series of patients with SPT [8,20,21,22,23,24,25,26,28,29,30,31,32,33,34].

Despite the rarity of such surgical procedures, standard pancreatic resections such as distal spleno-pancreatectomies or pancreaticoduodenectomies are widely considered safe in children, with almost nil mortality and relatively low severe morbidity rates [20,21,24,27,28,29,30,32,35,36,37]. Standard pancreatic resections represent the most frequent types of pancreatic resections used to treat pancreatic tumors in children—61.5% to 75% of the cases [8,25,27,30,31,32,34,38]. Overall and severe morbidity rates after standard pancreatic resections in children are 18.6–54.5% and 6.7–16.7%, respectively [8,21,25,27,28,30,31,33,35,37]. The most frequent source of surgical morbidity was postoperative pancreatic fistula (POPF)—8.6% to 20.5% of cases [8,21,25,27,28,30,31,33]. It is worth mentioning that the most significant part of studies comparing outcomes of pancreaticoduodenectomies in children and adults did not find any significant difference between the groups regarding the overall morbidity and mortality rates [32,35], except for an increased bile leak rate in children [32]. A study also reported no significant differences in morbidity and mortality rates between children and adults for distal pancreatectomies [32]. However, one study suggests better outcomes for pancreatectomies in children than in adults [27].

A multicentric study including 65 children with pancreaticoduodenectomies reported overall and severe morbidity rates of 18% and 10.8%, with a 2% in-hospital mortality rate; POPF was the primary source of morbidity—14% [17]. A recent single-center study including 73 pancreatectomies in children reported overall morbidity rates of 34.2%, with severe morbidity rates of 11% for pancreaticoduodenectomies and 17% for distal spleno-pancreatectomies; the 90-day mortality was 0% [27].

Duodenum-preserving pancreatic head resection is an organ-sparing alternative to pancreaticoduodenectomy for a specific pathology, with fewer early postoperative complications [39]. Benign and low-grade malignant pancreatic pathologies represent the indications for such a conservative surgical procedure, with chronic pancreatitis as a leading indication; pancreatic malignancies are widely considered a contraindication [39,40]. Duodenum-preserving pancreatic head resection is an exceptional surgical procedure in children compared with adults. A few studies, including 3 to 25 cases, have reported duodenum-preserving pancreatic head resection in children [22,27,41,42]. However, in two of the studies mentioned above, which pathologies were treated with such a procedure was not reported [22,27,42], while in the other two studies, only SPT patients were included [41,42]; in two studies SPT, neuroendocrine tumors, trauma, and other benign conditions were the indications [43,44]. Nevertheless, as in adults, chronic pancreatitis and benign and low-grade malignant pathology are considered indications for such a procedure in children; at the same time, for PB or other pancreatic malignancies, a duodenum-preserving pancreatic head resection is considered oncologically unsafe [22,27,42,43,44]. To our knowledge, no children with duodenum-preserving pancreatic head resections for PB have been reported in the literature. Only one study compares duodenum-preserving pancreatic head resection and pancreaticoduodenectomies in children, showing similar early postoperative complication rates [44].

In the last few years, a minimally invasive approach has been safely introduced in clinical practice for a few types of pancreatectomies in children for a specific pathology, and it was also associated with very low morbidity rates [45,46]. However, a review published in 2022 identified only 77 children with minimally invasive pancreatic surgery in the literature [46]. A few studies comparing open and laparoscopic distal pancreatectomies for SPT in children identified shorter hospitalization and better recovery after the laparoscopic approach [47,48].

Spleen-preserving distal pancreatectomies have been introduced in clinical practice for a specific tumoral pathology as an alternative to classical distal spleno-pancreatectomies to overcome the potential negative consequences of splenectomies—this issue particularly interests children, where spleen preservation is even more important than adults. Preserving the spleen during distal pancreatectomies has been associated with reduced rates of postoperative infectious complications in a few adult studies [49]. In contrast, other recent studies of adult patients found no significant differences [50]. Splenectomy may lead to substantial risks such as infections, thromboembolic events, pulmonary hypertension, cancer, or other complications [51]. A few studies have reported 7% to 8% rates of post-splenectomy sepsis in children [52,53]; children with splenectomy require particular attention and management to prevent infections [54]. Interestingly, with the advent of the laparoscopic approach, a recent study has reported high rates of venous thromboembolism after elective laparoscopic splenectomies—0.41%, double compared with other laparoscopic abdominopelvic procedures in children [55]. In this context, preserving the spleen in children is paramount and should be the first option when performing distal pancreatectomies.

A relatively extensive experience from the Asan Medical Center with 28 children with spleen-preserving distal pancreatectomies for SPT has shown overall and severe morbidity rates of 57.1% and 10.7%, respectively, and a nil mortality rate. The most frequent complication was POPF, a clinically relevant one in 10.7% of the patients. It is worse to mention that the most significant part of the patients in the Korean cohort have had a minimally invasive approach (85.7%), and no differences in outcomes were observed between the splenic vessel preservation and splenic vessel resection techniques [26]. A Chinese study including 104 children resected for different pancreatic tumors (70.2% SPT and 18.3% PB) has reported an 86.1% use of spleen-preserving distal pancreatectomies for tumors of the pancreatic tail, with 37.2% overall morbidity rates [22].

A central pancreatectomy is a rare type of pancreatectomy in adults and children, even at high-volume centers [56]. The first central pancreatectomy in a child was reported by Fisher and co-workers in 2007 [18]. However, to date, it appears that there are reported a number of 138 cases of central pancreatectomy in children, the main indications being represented by SPT [8,15,18,19,21,22,23,24,25,27,28,33,42,57,58,59,60,61] with 61.1–62.7% morbidity rates, with POPF as a leading cause [22,23,25]. The overall morbidity and POPF rates after central pancreatectomies for SPT vary between 0% and 100%, as shown in Table 1. A central pancreatectomy implies preservation of the spleen and normal pancreatic tissue; thus, it potentially has the lowest impact of all types of pancreatectomies on long-term pancreatic functions. A meta-analysis of adult patients comparing distal pancreatectomies with central pancreatectomies has shown significantly increased rates of complications (including severe ones and POPF) for central pancreatectomies but also significantly lower rates of both exocrine and endocrine insufficiency rates [56]. Furthermore, comparing central pancreatectomies with spleen-preserving distal pancreatectomies in adults, the loss of normal pancreatic parenchyma is significantly lower for central pancreatectomies [62]. Lower remnant pancreatic volume strongly correlates with postoperative new-onset diabetes in adults [63]. Considering the high morbidity rates of central pancreatectomy in adults, a tailored approach was recently proposed for selecting patients who would benefit from central pancreatectomy without jeopardizing the morbidity [64].

A systematic review of the literature addressing SPT and pancreatic malignancies in children published in 2018 (including 489 patients) reported using spleen-preserving distal pancreatectomies and central pancreatectomies in 20% of the cases and 9.6%, respectively [8].

Enucleation has also been proposed as an alternative to standard pancreatic resection in a few particular cases of pancreatic tumors in children to preserve better pancreatic functions [23]. However, compared with standard pancreatic resections or even central pancreatectomies, enucleation in children is associated with increased positive resection margins and clinically relevant POPF [23]. Interestingly, no significant differences were observed between the groups of children with enucleation and central pancreatectomies for the postoperative endocrine insufficiency rates [23]. In adults, pancreatic enucleation is recommended for benign and low-grade malignant pathology when surgery is indicated, and the procedure is feasible with no significant impact on the long-term quality of life in adults [65]. However, in adults, for pancreatic malignancies, enucleation is widely considered oncologically unsafe [65], albeit it may play a role in the therapy of particular malignancies, such as pancreatic metastases of renal cancer origin [66].

Morbidities after pancreatic resections in children are provided in Table 2.

## 3. The Burden of Long-Term Pancreatic Function Deficiencies after Pancreatectomies in Children

Data about long-term functional outcomes after pancreatectomies in children are scarce (Table 1 and Table 2).

A few studies have shown that the rate of exocrine and endocrine pancreatic insufficiency after pancreaticoduodenectomies in children is 10–100% and 8–40%, respectively [17,29,32,34,35,36,37,42,67]. Other studies, including standard and organ-sparing pancreatectomies for children, have identified 0% to 83.3% rates for pancreatic exocrine and 1% to 20% for pancreatic endocrine insufficiencies [22,27,31,34]. A few studies reported a 0% to 15% rate of new-onset diabetes and a 0% to 16% pancreatic exocrine insufficiency rate after distal pancreatectomies in children, with nil diabetes rates for enucleation and central pancreatectomy [23,32,34]. Two studies report nil exocrine and endocrine insufficiency rates after standard pancreatic resections in children [28,36].

Furthermore, steady growth and body mass index curves in the normal range were observed in 73.3% to 100% of children with standard pancreatic resections [28,31,34,67]. A study published in 1994 analyzing the long-term nutritional and metabolic consequences of pancreaticoduodenectomies in children found that the children could grow and develop normally if pancreatic enzymes and fat-soluble vitamins were provided [68]. In recent studies, vitamin deficiencies (particularly vitamin D) were reported in 5% to 62.5% of children with pancreaticoduodenectomies [17,34]. Nevertheless, a study reported a good quality of life in the long-term outcomes in more than 90% of the children after pancreaticoduodenectomies [67]. One might suggest that early identification of pancreatic deficiencies after pancreatectomies and their adequate treatment is essential to prevent nutritional deficiencies and allow normal growth in children [34].

Interestingly, a systematic review identified long-term postoperative exocrine and endocrine insufficiency rates of 20% and 4.3% in children with pancreaticoduodenectomies for pancreatic tumors; surprisingly, preservation of the pylorus during pancreaticoduodenectomies was associated with increased rates of pancreatic function deficiency compared with the classical Whipple [8].

However, the most significant part of data about late metabolic morbidity after pancreatectomies comes from an adult series of patients. Thus, in adults, after pancreaticoduodenectomies, the new-onset diabetes rates vary between 8% and 30%, while the pancreatic exocrine insufficiency rates vary between 25% and 49% [40,69]. Regarding distal pancreatectomies, in adults, the reported rates of postoperative endocrine and exocrine insufficiencies are 36% and between 18% and 80%, respectively. Furthermore, a high degree of pancreatic endocrine and exocrine insufficiency is associated with shorter survival [69].

Both distal spleno-pancreatectomies and pancreaticoduodenectomies imply the removal of approximately 50% of the pancreas. In contrast, in organ-sparing pancreatectomies such as spleen-preserving distal pancreatectomies and central pancreatectomies, the loss of normal pancreas is much lower. The occurrence of post-pancreatectomy dysfunctions depends on the extent of pancreatic resection and the functional quality of the pancreatic remnant. Remnant pancreatic volume is correlated with the risk of postoperative exocrine pancreatic insufficiency after standard pancreatic resections in adults [70]. Stenosis of the pancreatic-digestive anastomosis after pancreaticoduodenectomies might be another cause of pancreatic remnant atrophy with secondary exocrine and endocrine deficiencies in children. This situation is relatively common after pancreaticoduodenectomies in adult patients with long-term survival [29,67]. Furthermore, the rate of postoperative pancreatic functional deficiencies after standard pancreatectomies depends also on the reported timing after resection; thus, a study in adults has reported rates of exocrine and endocrine pancreatic insufficiencies beyond the 90-day point time after standard pancreatectomies of 16% and 43%, respectively [71]. Adjuvant chemotherapy was identified as an independent predictor of exocrine pancreatic insufficiency after standard pancreatic resections in adults [71].

A recent meta-analysis performed in adult patients has shown that standard pancreatic resections are associated with a considerable risk for new-onset diabetes and pancreatic exocrine insufficiencies, while parenchyma-sparing pancreatectomies are associated with low-grade metabolic dysfunctions [40]. Interestingly, a study published in 2005 by Berrocal and co-workers demonstrated normal age-dependent growth in a significant part of children with near-total (i.e., 90–95%) pancreatectomy due to pancreas regeneration [72]. However, another study reported 96% insulin-dependent diabetes and 72% exocrine insufficiency rates at 11 years following near-total pancreatectomies in children [73]. Nevertheless, children and adults may present different functional consequences after pancreatectomies. One nationwide multicenter study suggested that the development of endocrine and exocrine insufficiencies after pancreatic resections in children and young adults is substantially lower than in normal adults [32]. Similar conclusions were reached in another recent review, reflecting the more significant functional reserve of the pancreas in young patients [34].

Studies comparing duodenum-preserving pancreatic head resection with pancreaticoduodenectomies in adults reported better preservation of pancreatic functions and quality of life without increasing the local recurrence rates [39,40]. A meta-analysis performed in adult patients has shown statistically significantly lower rates of new-onset diabetes mellitus and pancreatic exocrine insufficiencies after duodenum-preserving pancreatic head resections, compared with the pancreaticoduodenectomies: 5% vs. 15.7%, and 6.7% vs. 44.3%, respectively. Furthermore, the rate of postoperative steatohepatitis after duodenum-preserving pancreatic head resection in adults was statistically significantly lower compared with pancreaticoduodenectomies: 3% vs. 23.8% [40]. One study comparing duodenum-preserving pancreatic head resection and pancreaticoduodenectomies in children showed similar recurrence rates but statistically significantly lower rates for late complications for duodenum-preserving pancreatic head resections (14.3% vs. 100%); interestingly, no differences were observed for both endocrine and exocrine insufficiency rates [44]. Another study, including a limited number of children, reported nil rates for postoperative diabetes and a 36% rate for pancreatic insufficiencies after duodenum-pancreatic head resection [43].

It is worth mentioning a recent systematic review assessing potential risk factors for developing diabetes mellitus after pancreatectomies in adults, showing new-onset diabetes rates between 9% and 24% after pancreaticoduodenectomies, between 3% and 40% after distal pancreatectomies and between 0% and 14% for central pancreatectomies [63]. Furthermore, the rate of pancreatic insufficiencies after pancreatectomies in adults depends not only on the extent of resection but also on preexisting diabetes mellitus or elevated preoperative fasting plasma glucose and pancreatic disease and texture (chronic pancreatitis and ductal adenocarcinoma being associated with an increased risk of both pre- and postoperative pancreatic insufficiencies) [63,74,75]. To our knowledge, no study has assessed such potential risk factors for children, as mentioned above.

## 4. Current Surgical Approach and Outcomes of PB in Children

The approach of a child diagnosed with PB is multimodal by multidisciplinary teams, and resection plays a pivotal role in obtaining good long-term outcomes. Although many children are initially unresectable at diagnosis [6,9,10,11,12,13,59,76], neo-adjuvant treatments, including primary chemotherapy, may lead to tumor downstaging and/or downsizing, and resection with negative resection margins is feasible in most patients [6,10,11,13,59]. A few studies have reported a resectability rate for PB in children of 72.7% to 81% [11,12]. Achievement of negative resection margins after PB pancreatectomies is crucial for disease-free and long-term survival [3,6,10,11,12,13].

Complete resection of PB in children in a multimodal approach, including sometimes neoadjuvant and/or adjuvant chemotherapy, is feasible in almost 80% of the cases [12], and it is associated with long-term survival. Thus, complete resection of PB is associated with a median survival of 39 to 53 months (5–336 months) [1,5,6,8,12,25,30,37,59,76,77,78,79,80,81], while the 5-year overall survival rate is 50–94.4% [6,7,9,11,12,59,80,82]. The recurrence rate after the resection of PB in children is about 14.7% to 26% [6,8,12], at a median time of 20 months after resection (1–39 months) [12]. Similar long-term survival for children and adults was reported in a Chinese study [76], while another study has shown significantly better survival in children than adults [12].

Although positive lymph nodes after resection for PB represent a poor prognostic factor, there is no consensus about the mandatory or extent of lymph node dissection [10,83]. Suspected positive lymph nodes at imaging examination should refer the patient for neo-adjuvant chemotherapy [10]. A recent consensus meeting has proposed loco-regional lymph node dissection only for patients with positive lymph nodes (enlarged lymph nodes on preoperative imaging and intraoperative positive frozen-section histopathological analysis) [10]. A few studies reported positive lymph nodes in 17% to 50% of the children resected for PB [82,83].

## 5. The Potential Role of Organ-Sparing Pancreatectomies in PB in Children

The first pancreaticoduodenectomy for PB in a child was made by Walter Becker in 1956 [84], and till 2010, only 27 pancreaticoduodenectomies for PB in children were reported [80]. Although most children resected for PB underwent standard pancreatic resections [2,4,5,6,8,13,59,61,80,83], a paradigm change has been observed in organ-sparing pancreatectomies in the last few years. Thus, in a recent Chinese study including 18 children with resected PB, organ-sparing pancreatectomies, including spleen-preserving distal pancreatectomies and central pancreatectomies, were performed in 50% of patients (spleen-preserving distal pancreatectomies—27.8%, central pancreatectomies—22.2%), while standard pancreatic resections such as distal spleno-pancreatectomies and pancreaticoduodenectomies were performed in the remaining 50% of the patients; no differences in recurrence rates or survival were observed between the group of children with standard and organ-sparing pancreatectomies for PB [59]. A systematic review published in 2018 reported that spleen-preserving distal pancreatectomies and central pancreatectomies were used in 26.5% and 11.8% of children resected for PB [8].

A few essential issues should be considered when approaching a pancreatic resection in a child diagnosed with PB. Thus, the type of resection should be guided by tumor stage, tumor location, and oncological safety (complete resection of the tumor with negative resection margins). An appropriate preoperative imaging evaluation of a child with PB is critical for proper staging and management; magnetic resonance imaging emerged as the first choice to assess a liver mass in children, including PB [10] (Figure 2). Considering the above-mentioned critical issues of a safe pancreatic resection for a child diagnosed with PB, standard pancreatic resections appear to be the first option for an oncologically safe operation, as a recent international guideline recommends [10]. However, considering the long-term survival of these children resected for PB, in the context of long life expectancy after pancreatectomies, preservation of the pancreatic functions also emerges as a critical issue. Thus, as previously highlighted, organ-sparing pancreatectomies have been used as oncological radical pancreatectomies in the last few years in patients with PB as an alternative to standard pancreatic resection, the later ones being widely considered to be associated with high rates of postoperative pancreatic dysfunctions.

A distal pancreatectomy is usually performed for PB in the pancreatic body, and guidelines recommend preserving the spleen [6].

A central pancreatectomy was initially proposed for benign and low-grade malignant tumors of the pancreas [56]. However, central pancreatectomies were proven oncologically safe in adults for a few malignancies, such as metastases of other neoplasms to the pancreas [85].

Dumitrascu and co-workers reported the first central pancreatectomy for PB in 2011 in a 16-year-old girl [58]; remarkably, the patient has had normal growth and is disease-free 15 years after resection with no clinical signs of exocrine insufficiency or diabetes. Since then, 11 other children have undergone central pancreatectomies for PB worldwide [8,25,59,61], showing complete removal of the tumor, but data about their long-term functional and survival outcomes are scarce (no long-term functional data; only three children have long-term survival data being alive, without recurrence at 40, 50, and, respectively, 74 months after central pancreatectomies) [59]. A central pancreatectomy has been proven oncologically safe for a child with Ewing sarcoma [15].

Enucleation for PB has been associated with high rates of positive resection margins [6]. As mentioned above, positive resection margins have a detrimental effect on disease-free and long-term survival [6,10,13], with incomplete resection being associated with only 28.5% of 5-year disease-free survival, compared with 75% of negative resections [6].

Many children resected for PB underwent chemotherapy: 53% to 100% of cases [6,12,61,79,80]; PB is widely considered sensitive to chemotherapy [10]. The first-line chemotherapy in children with PB is represented by the association of cisplatin and doxorubicin (PLADO regimen) in both neoadjuvant and adjuvant settings [10]. Chemotherapy was identified as an independent predictor of exocrine pancreatic insufficiency after standard pancreatic resections in adults [71], and a high degree of pancreatic endocrine and exocrine insufficiency is associated with shorter survival [69].

Diabetes, including one after pancreatectomies, increases the risk of mortality from cancer and morbidity from the treatment of cancer in adults, and a few platinum-based drugs may favor diabetes occurrence [86]. Endocrine complications and metabolic syndrome are highly prevalent as late effects in childhood cancer survivors [87,88], as children with complete resection for PB are. Nevertheless, children with diabetes are at high risk of developing several long-term complications, including micro- and macrovascular ones [89,90,91], and their treatment can be challenging [91].

The aspects mentioned above should also be considered in curative-intent surgery for PB. Thus, central pancreatectomies might mitigate the burden of postoperative pancreatic deficiencies and their consequences in particular cases in the pancreatic body (Figure 1 and Figure 2).

The feasibility of complete resection (i.e., with negative resection margins) in a child diagnosed with PB depends on the tumor location, size, and local extension [10]. Organ-sparing pancreatectomies such as spleen-preserving pancreatectomies or central pancreatectomies are an alternative to standard pancreatectomies for PB in children whenever a complete resection can be achieved (based on the factors mentioned above), also taking into consideration that a systematic lymph node dissection is not a standard approach for such pathology [10].

## 6. Conclusions

Complete surgical resection in the context of a multimodal approach has been associated with excellent long-term survival in children diagnosed with PB. Standard pancreatic resections, such as pancreaticoduodenectomies and distal spleno-pancreatectomies, may lead to significant long-term pancreatic functional deficiencies. Postoperative pancreatic functional deficiencies may interfere with children’s development, considering their long life expectancy and the significant role of pancreatic functions in their nutritional status and growth. Organ-sparing pancreatectomies are associated with better long-term pancreatic functional outcomes, particularly central pancreatectomies, and have a reduced impact on children’s development and quality of life without jeopardizing oncological safety. The long-term preservation of pancreatic functions should not be disregarded when performing pancreatectomies for PB in children. A subset of patients with PB might benefit from organ-sparing pancreatectomies, particularly from central pancreatectomies, with the same oncological results as standard pancreatectomies but with significantly less impact on long-term functional outcomes.

## Figures and Tables

**Table 1:** Summary of studies from the literature reporting early morbidity and late functional outcomes of central pancreatectomies for a specific tumoral pathology in children.

Author, Year	No of Patients	Overall Morbidity	POPF	Impaired Pancreatic Function	Follow-Up Time (Months)
**SPT**					
Fisher, 2007 [18]	1	0%	0%	0%	10
Sokolov, 2009 [57]	1	0%	0%	NR	24
Muller, 2012 [28]	2	50%	50%	E—0%, Ex—50%	50
Nachulewicz, 2015 [19]	1	100%	100%	NR	36
Crocoli, 2018 [21]	4	0%	0%	0%	NR
Cho, 2019 [23]	51	NR	63%	E—3.9%	442
van Ramshorst, 2021 [60]	1	100%	100%	0%	NR
Jones, 2021 [33]	1	100%	100%	NR	13
Jentzsch, 2023 [42]	1	100%	0%	0%	5
**PB**					
Dumitrascu, 2011 [58]	1	0%	0%	0%	180

SPT—solid pseudopapillary tumor; PB—pancreatoblastoma; POPF—postoperative pancreatic fistulae; E—endocrine insufficiency; Ex—exocrine insufficiency; NR—not reported.

**Table 2:** Summary of relevant studies from the literature reporting early and long-term outcomes of pancreaticoduodenectomies, duodenum-preserving pancreatic head resections, and distal pancreatectomies for pancreatic tumors in children.

Author, Year	No of pts	Overall Morbidity	POPF	Endocrine Insufficiency	Exocrine Insufficiency	Median Follow-Up Time (Months)
**Pancreaticoduodenectomies**						
Dasgupta, 2005 [35]	5	40%	NR	NR	NR	NR
Muller, 2012 [28]	11	54.5%	9.1%	NR	NR	NR
D’Ambrosio, 2014 [36]	5	0%	0%	20%	0%	24
Park, 2016 [67]	10	NR	NR	10%	30%	126
Lindholm, 2017 [37]	12	50%/16.7% ^a^	8.3%	0%	83.3%	NR
Scandavini, 2017 [31]	5	40%	20%	0%	0%	80
Huang, 2019 [59]	6	NR	NR	NR	NR	NR
Qin, 2019 [44]	6	16.7%	0%	0%	0%	31
Vasudevan, 2020 [17]	65	16.9%/12.3% ^a^	13.8%	13.8%	29.2%	45.6
Jones, 2021 [33]	9	NR	11%	NR	NR	NR
Bolasco, 2021 [34]	8	NR	NR	25%	50%	83.4
Fuchs, 2023 [27]	19	11% ^a^	NR	NR	NR	NR
**Duodenum-preserving pancreatic head resections**						
Snajdauf, 2019 [43]	21	9.5%/4.8% ^a^	NR	0%	36%	115.2
Qin, 2019 [44]	22	63.6%	50%/31.8% ^b^	0%	4.5%	31
Fuchs, 2023 [27]	10	0%	NR	NR	NR	NR
**Distal spleno-pancreatectomies**						
Huang, 2019 [59]	3	NR	NR	NR	NR	NR
Bolasco, 2021 [34]	3	NR	NR	0%	0%	30
**Spleen-preserving distal pancreatectomies**						
Huang, 2019 [59]	5	NR	NR	NR	NR	NR
Bolasco, 2021 [34]	4	NR	NR	0%	0%	61.2
Kwon, 2022 [26]	28	57.1%/10.7% ^a^	89.3%/10.7% ^b^	NR	NR	NR
**Enucleations**						
Scandavini, 2017 [31]	3	33.3%	33.3%	0%	0%	80
Cho, 2019 [23]	15	NR	66.7%	0%	0%	746.8
Qin, 2019 [44]	7	57.1%	28.6% ^b^	0%	0%	31

^a^ Severe morbidity (i.e., >grade 2 Clavien–Dindo); ^b^ grade B–C; POPF—postoperative pancreatic fistulae; NR—not reported.

## Data Availability

All relevant data are provided in the manuscript.
